# Ice‐binding proteins confer freezing tolerance in transgenic *Arabidopsis thaliana*

**DOI:** 10.1111/pbi.12592

**Published:** 2016-07-14

**Authors:** Melissa Bredow, Barbara Vanderbeld, Virginia K. Walker

**Affiliations:** ^1^ Department of Biology Queen's University Kingston ON Canada; ^2^ Department of Biomedical and Molecular Sciences and School of Environmental Studies Queen's University Kingston ON Canada

**Keywords:** *Lolium perenne*, antifreeze, ice‐binding proteins, ion leakage, freezing survival, *Arabidopsis thaliana*

## Abstract

*Lolium perenne* is a freeze‐tolerant perennial ryegrass capable of withstanding temperatures below −13 °C. Ice‐binding proteins (IBPs) presumably help prevent damage associated with freezing by restricting the growth of ice crystals in the apoplast. We have investigated the expression, localization and *in planta* freezing protection capabilities of two *L. perenne* IBP isoforms, *Lp* IRI2 and *Lp* IRI3, as well as a processed IBP (*Lp* AFP). One of these isoforms, *Lp* IRI2, lacks a conventional signal peptide and was assumed to be a pseudogene. Nevertheless, both *LpIRI2* and *LpIRI3* transcripts were up‐regulated following cold acclimation. *Lp* IRI2 also demonstrated ice‐binding activity when produced recombinantly in *Escherichia coli*. Both the *Lp* IRI3 and *Lp* IRI2 isoforms appeared to accumulate in the apoplast of transgenic *Arabidopsis thaliana* plants. In contrast, the fully processed isoform, *Lp* AFP, remained intracellular. Transgenic plants expressing either *LpIRI2* or *LpIRI3* showed reduced ion leakage (12%–39%) after low‐temperature treatments, and significantly improved freezing survival, while transgenic *LpAFP*‐expressing lines did not confer substantial subzero protection. Freeze protection was further enhanced by with the introduction of more than one IBP isoform; ion leakage was reduced 26%–35% and 10% of plants survived temperatures as low as −8 °C. Our results demonstrate that apoplastic expression of multiple *L. perenne* IBP isoforms shows promise for providing protection to crops susceptible to freeze‐induced damage.

## Introduction

Many overwintering temperate plants are susceptible to freeze injury during the coldest months. At subzero temperatures, ice crystals form in intracellular spaces, or the apoplast, creating an osmotic gradient that can result in cellular dehydration, expansion‐mediated lysis of plasma membranes and even death of the plant (Thomashow, 1998). In order to better adapt to freezing temperatures, some overwintering plants induce the expression of a family of protective proteins, designated ice‐binding proteins (IBPs). IBPs are members of a highly diverse family of proteins that have been identified in certain organisms including fish (Davies and Hew, 2013), insects (Duman, 2001), bacteria (Gilbert *et al*., 2004) and plants (Sidebottom *et al*., 2000). The activity of IBPs stems from their ability to irreversibly adsorb to ice crystals, resulting in the ‘shaping’ of ice as they become incorporated into the ice crystal lattice (Bar‐Dolev *et al*., 2012). IBPs are known to enhance freezing tolerance through two distinct properties: ice recrystallization inhibition (IRI), which prevents the growth of ice crystals at high subzero temperatures (Sandve *et al*., 2008), and thermal hysteresis (TH), or the depression of the freezing point in relation to the equilibrium melting point (Raymond and DeVries, 1977). While freeze‐avoidant organisms produce IBPs that depress the freezing point by several degrees, often referred to as antifreeze proteins (AFPs) (Davies and Hew, 2013; Duman, 2001), plants encode IBPs with low TH activity and rely on restricting ice crystal growth as a primary survival strategy (Sandve *et al*., 2011).

Although IBPs are not commonly found in plants and are absent in *Arabidopsis thaliana* for example, they have been identified and purified from more than a dozen plants including bittersweet nightshade (*Solanum dulcamara*) (Huang and Duman, 2002), carrot (*Daucus carota*) (Smallwood *et al*., 1999), winter rye (*Secale cereale*) (Hon *et al*., 1995) and perennial ryegrass (*Lolium perenne*) (Pudney *et al*., 2003). As ice crystal growth is commonly propagated in the apoplast, secretion of IBPs from the cytoplasm would prevent the recrystallization of extracellular ice, protecting cells from the effects of freeze‐induced cellular dehydration. Thus, it is not surprising that most of the IBPs that have been studied have been recovered from the apoplastic extracts of cold‐acclimated leaf tissue (Antikainen and Griffith, 1997; Griffith *et al*., 1992; Hon *et al*., 1994; Marentez *et al*., 1993). The presence of an N‐terminal signal peptide in most IBPs suggests secretion through the endoplasmic reticulum (ER) secretory pathway. An IBP from *S. dulcamara* has been reported to lack a signal peptide and remains intracellular (Huang and Duman, 2002), suggesting that IBPs might also function to prevent damage associated with intracellular ice nucleation.

The freeze‐tolerant perennial grass, *L. perenne* (*Lp*), is native throughout Europe and Eastern Asia where it can survive at temperatures as low as −13 °C (Thomas and James, 1993). Their IBPs have been termed ice recrystallization inhibition proteins (*Lp*IRIPs) due to their low TH activity (~0.3 °C at 1 mg/mL) (Lauersen *et al*., 2011), but relatively high IRI activity (Sidebottom *et al*., 2000). Four *Lp*IRIPs have been identified in *L. perenne*:* Lp*IRI1, *Lp*IRI2, *Lp*IRI3 and *Lp*IRI4 (Sandve *et al*., 2008). A partial protein product, named *Lp*AFP*,* was identified in another *L. perenne* cultivar (Sidebottom *et al*., 2000) and has been the subject of much *in vitro* characterization (Lauersen *et al*., 2011; Middleton *et al*., 2009).

*Lp*IRIPs have two distinct domains: a leucine‐rich repeat (LRR) domain and a carboxyl (C)‐terminal IRI domain, which consists of a series of repeated ‘ice‐binding’ motifs (NXVXG/NXVXXG, where X represents an outward‐facing residue). The IRI domain of these proteins has been predicted to fold into a β‐helix and the crystal structure of *Lp*AFP has verified this fold (Middleton *et al*., 2009). Specific residues on the ice‐binding face (IBF) allow *Lp*IRIPs to adsorb to ice crystals on the basal and primary prism planes resulting in hexagonal bipyramidal crystals (Kumble *et al*., 2008). N‐terminal to the IRI domain is a varying number of LRR motifs, likely derived from phytosulfokine LRR receptor tyrosine kinase sequences (Sandve *et al*., 2008). The retention of the LRR domain in three of the IBP isoforms may simply have allowed for the presence of the N‐terminal signal peptide for secretion to the apoplast.

Notably, one isoform, *Lp*IRI2, lacks most of the region upstream of the IRI domain, including an identifiable signal sequence, and likely evolved through duplication of *Lp*IRI4 and the subsequent deletion of the N‐terminal domain (Sandve *et al*., 2008). The absence of a signal peptide has led to the hypothesis that *LpIRI2* is a pseudogene. However, the modest sequence divergence following duplication of *LpIRI4* suggests that the gene may still be under selective pressure and thus retain some function (Sandve *et al*., 2008). Therefore, whether *LpIRI2* is a pseudogene, acts as an intracellular IBP or is secreted via a nonclassical pathway is not known.

Biotechnological applications of IBPs for the enhancement freeze tolerance have been tested in tobacco (*Nicotiana tabacum*) (Holmberg *et al*., 2001), potato (*Solanum tuberosum*) (Wallis *et al*., 1997) and tomato (*Solanum lycopersicum*) (Hightower *et al*., 1991). Generally, such transgenic plants have shown IBP accumulation in the apoplast, but few efforts have reported significant differences in freezing tolerance as shown by 50% lethality (LT_50_) assays. An exception is the enhanced freeze survival following the transfer of IBP sequences to *Arabidopsis thaliana* (Zhang *et al*., 2010). However, these studies have focused on the expression of a single IBP from a protein family, and we considered that the isoforms could work synergistically or cumulatively to restrict ice crystal growth. As well, we were particularly interested in determining whether *Lp*IRI2 has retained ice‐binding activity and thus function *in planta*, despite the loss of the N‐terminal signal peptide. We have now addressed these outstanding concerns through the expression of various *Lp*IRIP isoforms in *A. thaliana*, alone and in combination, in order to provide insight into the mechanisms underlying IBP‐mediated freezing tolerance.

## Results

### Bioinformatics analysis of *Lp*IRIP isoforms

**Figure 1:**
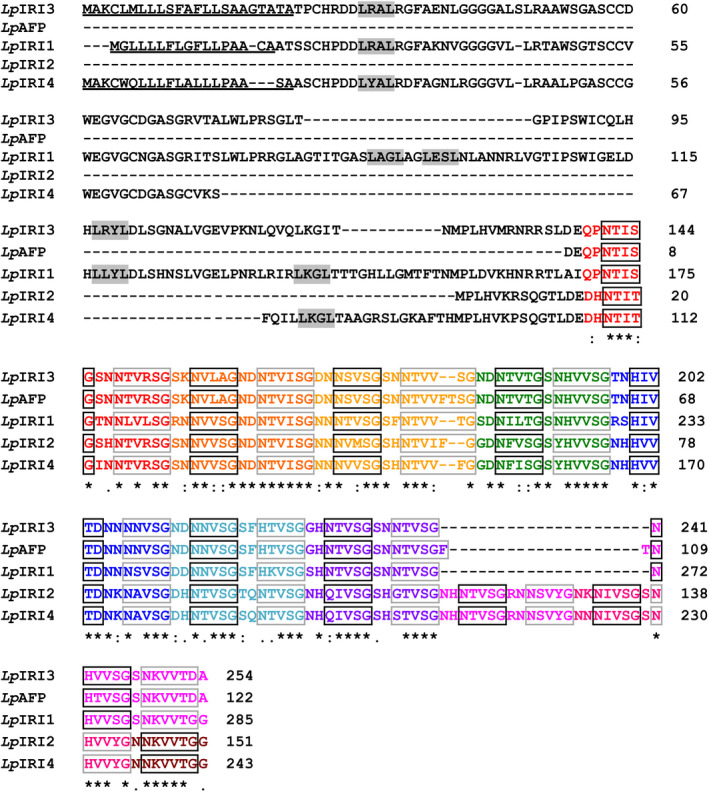
Amino acid sequence alignment of *Lp* IRIP sequences. *Lp* IRI3 ( EU 680850), *Lp* AFP ( AJ 277399), *Lp* IRI1 ( EU 680848), *Lp* IRI2 ( EU 680849) and *Lp* IRI4 ( EU 680851) were aligned using the ClustalW2 multiple sequence alignment tool. Putative signal peptides, predicted using the SignalP 4.1 server, are underlined. Leucine‐rich repeat motifs (LXXL) are in grey‐filled boxes and the amino acids corresponding to the predicted ice‐binding and non‐ice‐binding faces are in black and grey boxes, respectively. Each β‐helical turn, predicted by the Phyre2 algorithm, is identified by a distinct colour. (*) denotes a single, fully conserved reside; (:) denotes conservation between groups of highly similar properties (scoring > 0.5 in the Gonnet PAM 250 score), and (.) denotes conservation between groups of weakly similar properties (scoring =<0.5 in the Gonnet PAM 250 score).

Amino acid alignment of *Lp*IRIP isoforms shows high conservation of the C‐terminal residues, while the N‐terminal domains are more divergent (Figure 1). The N‐terminal domains of *Lp*IRIP isoforms have retained few LRR motifs (0–5 motifs across isoforms), with some isoforms having large deletions, or in the case of *Lp*IRI2, having not retained this domain all together. An N‐terminal signal peptide was identified for *Lp*IRI1, *Lp*IRI3 and *Lp*IRI4; however, as previously reported (Sandve *et al*., 2008), there is no apparent secretion signal within *Lp*IRI2 or *Lp*AFP amino acid sequences. Putative ice‐binding amino acids appear to be conserved across four sequences with the unprocessed proteins ranging in size from 151 to 285 residues. The Phyre2 algorithm predicted that all proteins would fold into a right‐handed β‐helix. While the *Lp*IRI3, *Lp*AFP and *Lp*IRI1 isoforms were predicted to fold into secondary structures with eight β‐helical loops, *Lp*IRI2 and *Lp*IRI4 were predicted to have ten loops, as the result of three additional ice‐binding motifs (Figure 1).

### Functional activity and transcript analysis of *Lp*IRIP sequences

**Figure 2:**
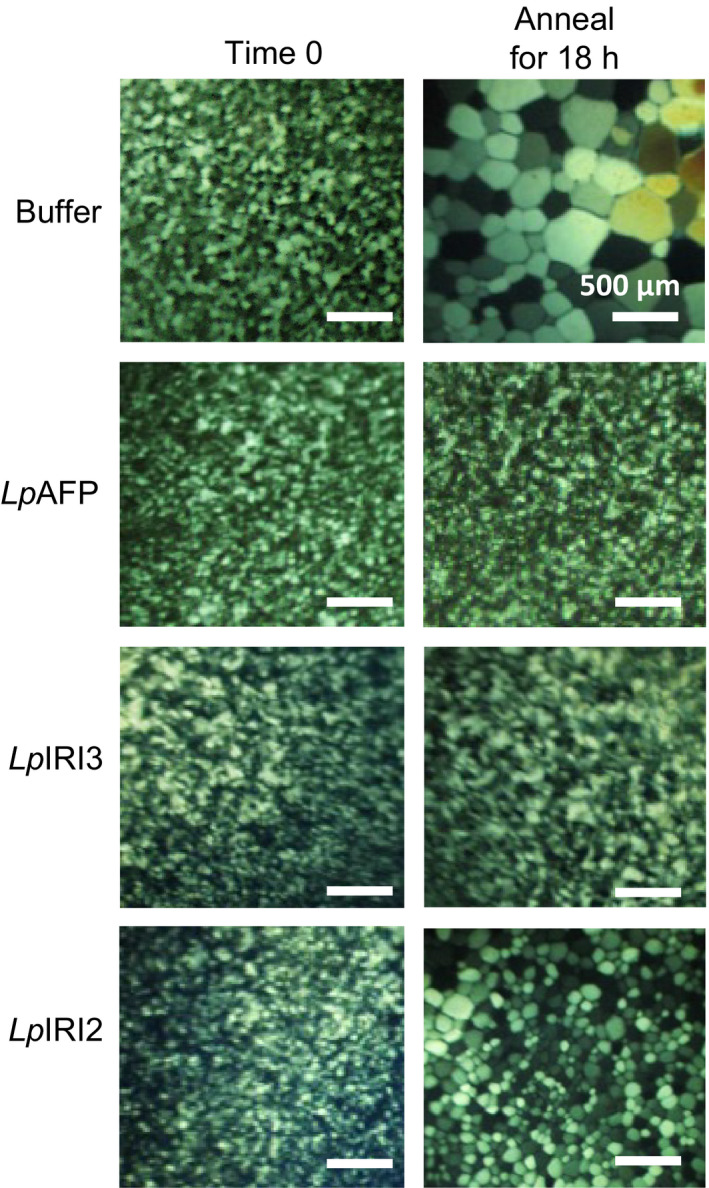
Ice recrystallization inhibition assessment of recombinantly produced *Lp* IRIPs. Purified *Lp* AFP,* Lp* IRI3 and *Lp* IRI2 proteins were used for a splat assay by diluting samples to a concentration of 0.01 mg/mL and holding ice crystals at −4 °C for 20 h. Shown here are representative images from triplicate assays.

When constructs encoding *Lp*IRI2 and *Lp*IRI3 isoforms were expressed in *E. coli,* all purified, recombinantly produced proteins restricted ice crystal growth in a splat assay (Figure 2). However, not all recombinant isoforms were equally effective at IRI. High levels of activity were seen with *Lp*AFP and *Lp*IRI3 at 0.01 mg/mL; however, *Lp*IRI2 only demonstrated mild IRI activity, with some ice crystal growth at the same concentration.

**Figure 3:**
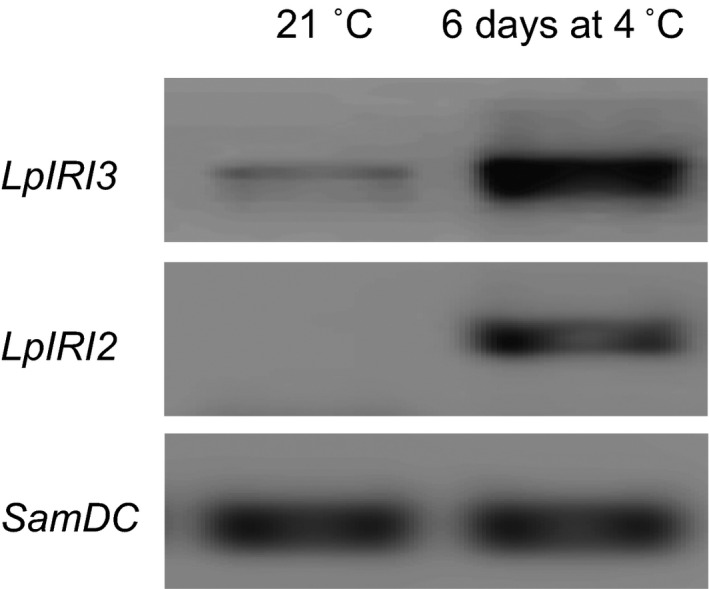
Reverse transcription PCR analysis of endogenous *LpIRI3 and LpIRI2* transcript levels. RNA was collected from the leaves of *L. perenne* grown at 21 °C or cold‐acclimated for 6 days at 4 °C. The *SamDC* transcript was used as a reference loading control. Experiments were conducted in triplicate.

Endogenous *L. perenne* transcript analysis generated ~500‐bp and ~850‐bp amplification products for *LpIRI2* and *LpIRI3*, respectively. Low levels of *LpIRI3* transcript were produced following incubation at 21 °C, but there was no evidence of the *LpIRI2* transcript (Figure 3). However, following a 6‐d cold acclimation (CA) period at 4 °C both *LpIRI3* and *LpIRI2* transcripts were abundant.

### Ice‐binding activity and localization of *Lp*IRIPs in *A. thaliana*

**Figure 4:**
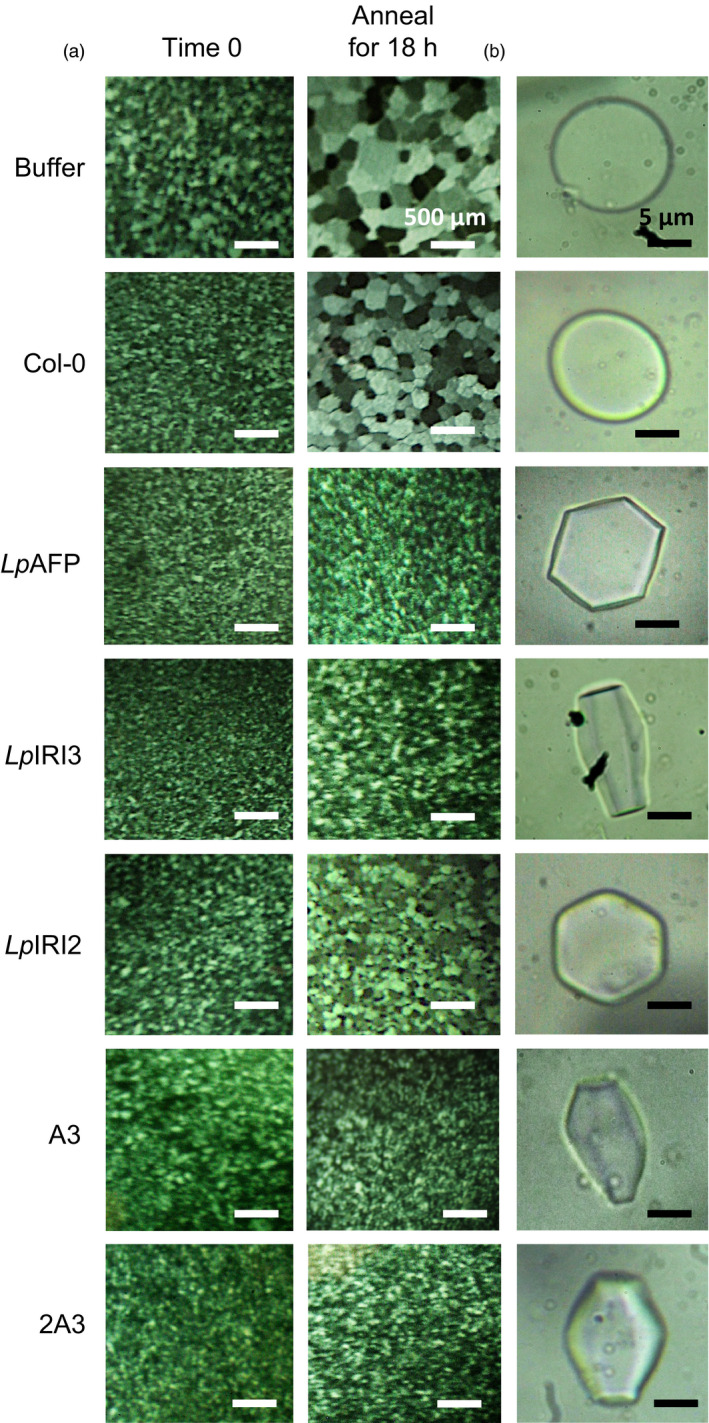
Ice‐binding phenotypes in transgenic *A. thaliana*. Ice recrystallization inhibition analysis of crystals annealed at −4 °C for 18 h (a) and ice crystal morphologies (b) using crude cell lysates collected from control (Col‐0) and transgenic plants, including lines expressing *LpAFP* and *LpIRI3* (A3) and all three sequences, *LpIRI2*,* LpAFP* and *LpIRI3* (2A3). A total protein concentration of 0.1 mg/mL was used for all assays. Only one representative sample is shown for each of the four *Lp* IRIP‐expressing lines. All experiments were performed in triplicate.

Following CA, crude cell extracts taken from all four independently generated *A. thaliana* lines transgenic for each of *LpAFP*,* LpIRI2*,* LpIRI3, LpAFP* and *LpIRI2* (designated A3), and all three *Lp*IRIP sequences (designated 2A3) showed functional ice‐binding activity as demonstrated by IRI analysis (Figure 4a). There was no IRI activity in control CA plants, and very low activity in those transgenic plants kept at room temperature prior to assay (not shown). This was expected as, at least for *Lp*AFP, circular dichroism‐monitored conformational changes to the β‐helical structure at temperatures >16 °C were coincident with a loss of activity (Lauersen *et al*., 2011). All CA *LpIRI2*‐transformed lines consistently showed IRI, but they had lower activity than *LpAFP*‐ and *LpIRI3*‐expressing lines (Figure 4a). Extracts from the transgenic plants expressing *LpIRI2* and *LpAFP* demonstrated hexagonal ice shaping indicative of adsorption to the primary prism plane (Figure 4b). Notably, *LpIRI3*, A3 and 2A3 transgenic lines showed hexagonal bipyramidal ice shaping, which is seen with more active AFPs that bind both the primary prism and basal planes. TH activity depends on both ice‐binding properties and protein concentration. Indeed, TH values obtained from cell lysates of our transgenic plants were similar for all the single expression lines with the multiple A3 and 2A3 lines showing greater activity levels (Table 1a).

**Table 1:** Thermal hysteresis values obtained from crude cell extracts (a) of transgenic lines expressing individual *LpIRIP* sequences, *LpAFP* and *LpIRI3* (A3), and all three sequences (2A3), compared with wild‐type *A. thaliana* (Col‐0), as well as apoplast extracts (b) of *mOrange*‐tagged *LpIRIP* lines compared with wild‐type *A. thaliana* plants and plants expressing *mOrange* alone

Transgenic line	Thermal hysteresis activity (°C)
(a)	
Col‐0	0
*LpAFP*	0.11
*LpIRI2*	0.08
*LpIRI3*	0.13
A3	0.15
2A3	0.18
(b)	
Col‐0	0
*mOrange*	0
*mOrange‐LpAFP*	0
*LpIRI2‐mOrange*	0.035
*LpIRI3‐mOrange*	0.07

**Figure 5:**
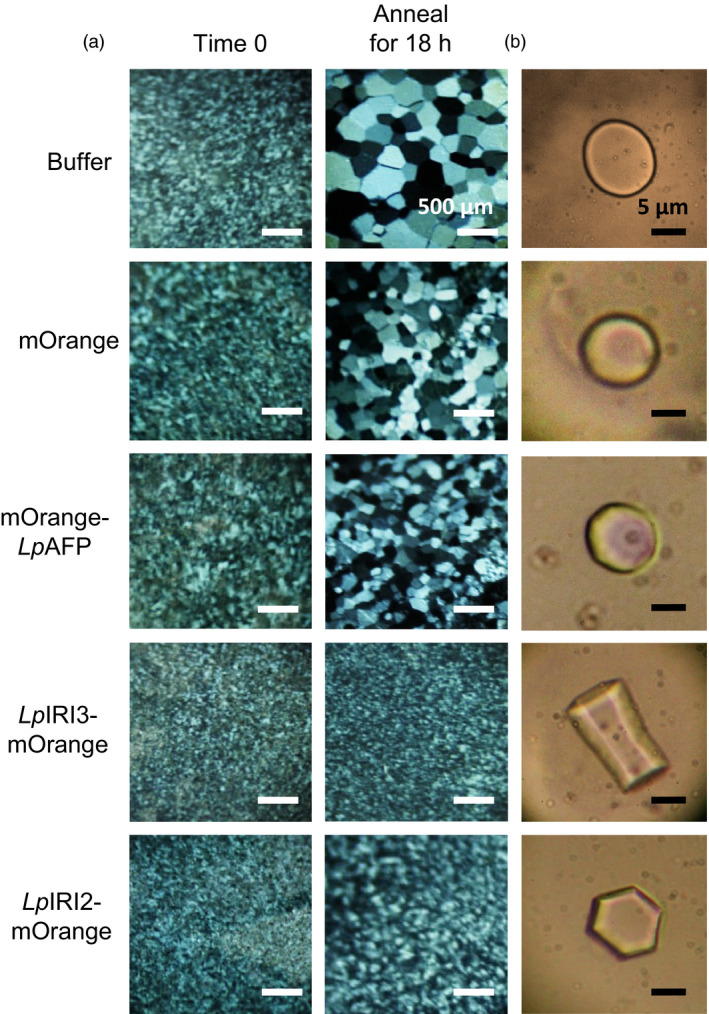
Ice‐binding phenotypes in apoplast extracts collected from transgenic *A. thaliana* plants bearing fluorescently tagged *Lp* IRIP sequences. Shown are the ice crystals seen during ice recrystallization inhibition analysis following an 18‐h annealing period at −4 °C (a) and the corresponding ice crystal morphologies (b). The apoplast extracts of transgenic lines expressing fluorescently tagged *Lp* IRIP constructs were compared to control (Col‐0) nontransgenic plants, at a total protein concentration of 0.1 mg/mL. Only one representative sample is shown for each *Lp* IRIP‐expressing line. Both assays were conducted in duplicate.

**Figure 6:**
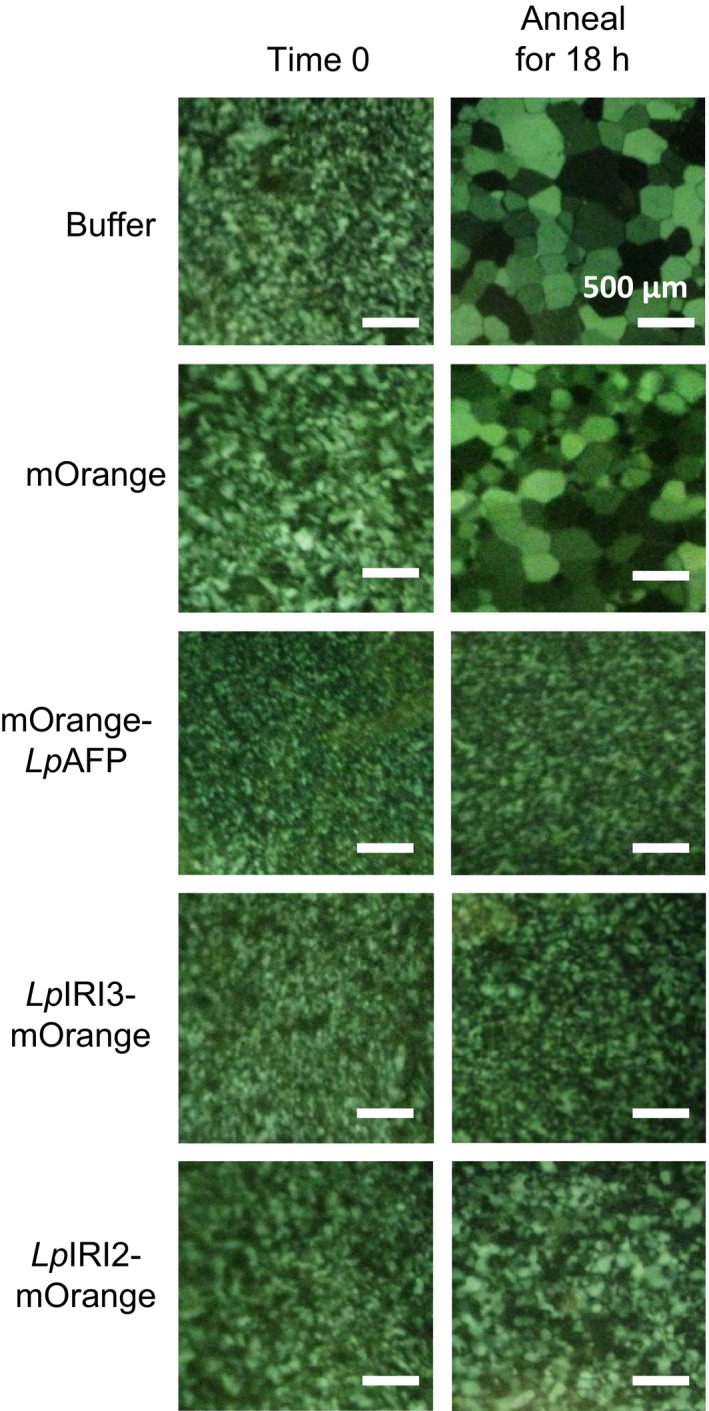
Ice recrystallization inhibition (IRI) analysis of plants expressing fluorescently tagged *Lp* IRIPs. Crude cell extracts were annealed at −4 °C for 18 h. Samples were assayed at a total protein concentration of 0.1 mg/mL. Only one representative sample is shown for each transgenic line. Assays were performed in triplicate.

**Figure 7:**
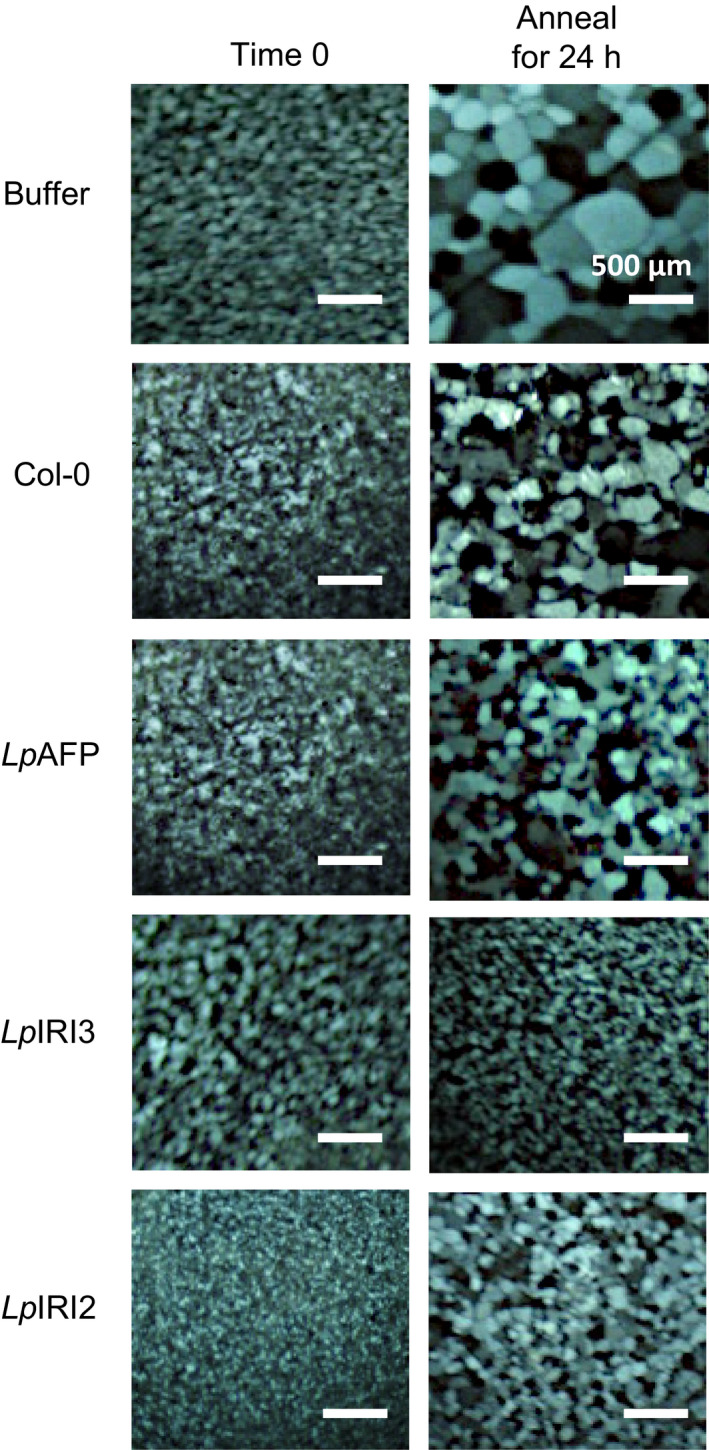
Ice recrystallization inhibition analysis of guttation fluid collected from transgenic *Lp* IRIP‐expressing *A. thaliana* plants. Guttation fluid collected from *LpAFP‐*,* LpIRI3‐* and *LpIRI2*‐expressing lines was used for a splat assay. Samples were held at −4 °C for 24 h and compared to nontransgenic control plants (Col‐0). A total protein concentration of 0.1 mg/mL was used for all experiments. Only one representative sample is shown for each *Lp* IRIP‐expressing line. Assays were conducted in triplicate.

In order to localize IBP activity, the ORFs of *Lp*IRIPs fused to fluorescent markers were expressed in *A. thaliana*. Confocal microscopy confirmed protein expression in all *LpAFP*,* LpIRI2* and *LpIRI3* transgenic lines. It was difficult to resolve cytoplasmic and apoplastic locations however, due to the large vacuoles in leaf and root tissues (Figure S1). Nevertheless, apoplastic extracts of transgenic *A. thaliana* lines expressing *LpIRI2‐mOrange* and *LpIRI3‐mOrange* showed fluorescence and had high levels of IRI activity (Table 2 and Figure 5a). Additionally, *LpIRI2‐mOrange* lines consistently showed hexagonal, primary prism plane ice shaping and *LpIRI3‐mOrange* lines exhibited hexagonal bipyramidal crystals, consistent with basal and primary prism plane adsorption (Figure 5b). Both *LpIRI2‐mOrange* and *LpIRI3‐mOrange* lines also demonstrated TH activity (Table 1b). In contrast, apoplastic extracts from *mOrange‐LpAFP* lines had similar levels of ice‐binding activity to that of *mOrange* transgenic controls, with low levels of fluorescence (Table 2), no observable IRI activity, ice‐shaping activity or TH activity (Figure 5 and Table 1b). To determine whether the *mOrange* tag had disrupted the ice‐binding activity of *Lp*IRIPs, the crude cell extracts of transgenic plants were assayed for IRI activity (Figure 6). While *mOrange*‐expressing lines had no ice crystal inhibition, all *Lp*IRIP‐expressing lines retained high levels of IRI activity. Investigations with guttation fluid of transgenic *Lp*IRIP‐expressing lines revealed similar results to the apoplastic extracts: transgenic *LpAFP‐*expressing *A. thaliana* and control plants had no detectable IRI activity with *LpIRI3* and *LpIRI2* lines with high levels of IRI activity (Figure 7). It should be noted, however, that the activity observed in *LpIRI2*‐expressing lines was less than in guttation fluid obtained from *Lp*IRI3 lines (Figure 7).

**Table 2:** Mean fluorescence readings (wavelength emission and excitation of 562 nm and 548 nm, respectively) obtained using apoplast extracts (0.1 mg/mL total protein; triplicate samples) of *mOrange‐*tagged *Lp*IRIP‐expressing *A. thaliana* lines (as described in Table 1)

Transgenic line	Relative fluorescence units (RFUs)
*mOrange*	1037
*mOrange‐LpAFP*	1100
*LpIRI2‐mOrange*	12 834
*LpIRI3‐mOrange*	13 474

Values were normalized based on the level of fluorescence emitted in control, nontransgenic lines.

### Electrolyte leakage in transgenic leaves

**Figure 8:**
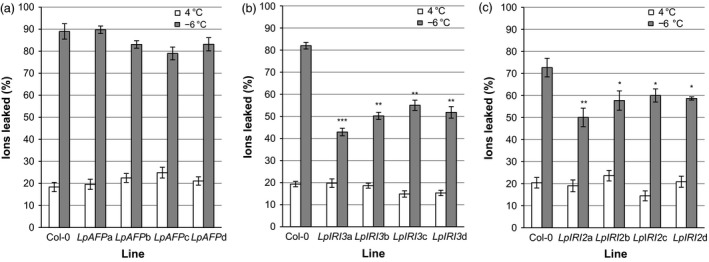
Ion leakage produced by the leaves of transgenic *Lp* IRIP‐expressing *A. thaliana* lines. Shown are the results of *A. thaliana* plants expressing *LpAFP* (a), *LpIRI3* (b) and *LpIRI2* (c) constructs following incubation at 4 °C or freezing to −6 °C. Transgenic plants were compared to nontransgenic *A. thaliana* plants (Col‐0). Ion leakage is represented as the proportion of ions leaked following treatment in relation to the total number of ions in the leaf sample. All experiments were performed in triplicate (*n* = 12). Error bars represent standard errors of the mean and asterisks denote a significant reduction in ion leakage compared with controls (*P < 0.05, **P < 0.005, ***P < 0.0005; two‐tailed *t*‐tests).

When leaves were collected from CA transgenic *A. thaliana* plants and incubated at temperatures slowly ramped down to −6 °C, freezing resulted in the leakage of ions, as assessed by conductivity. The expression of *LpAFP* in transgenic plants appeared to reduce leaf ion leakage by 6%−10% in three of the four independent lines, but these values were statistically insignificant when compared to control plants (Figure 8a). In contrast, expression of *LpIRI3* and *LpIRI2* decreased ion leakage by 30%−39% and 12%−22%, respectively (Figure 8b and c).

**Figure 9:**
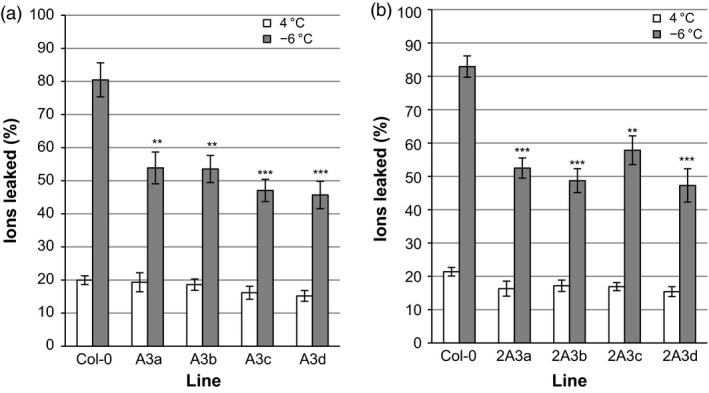
Ion leakage produced by the leaves of transgenic *A. thaliana* lines expressing multiple *Lp* IRIPs. Plants expressing *LpAFP* and *LpIRI3* (A3) (a) and lines transformed with *LpIRI2*,* LpAFP* and *LpIRI3* (2A3) (b) were compared to nontransgenic *A. thaliana* (Col‐0) plants. Leaves of transgenic and control *A. thaliana* plants were incubated at 4 °C or frozen to −6 °C prior to measuring ion leakage, which is represented as the proportion of ions leaked following treatment in relation to the total number of ions in the leaf sample. All experiments were performed in triplicate (*n* = 12 for each experiment). Error bars represent standard errors of the mean and asterisks denote a significant reduction in ion leakage compared with control plants **P < 0.005, ***P < 0.0005; two‐tailed *t*‐tests).

Transgenic plants expressing multiple *Lp*IRIPs also showed reduced electrolyte leakage compared with controls. *A. thaliana* leaves from plants expressing A3 constructs showed a 28%−35% decrease in electrolyte leakage following a −6 °C treatment (Figure 9a). Transgenic 2A3 lines similarly showed a 26%−35% reduction in electrolyte leakage (Figure 9b).

### *Lp*IRIPs and *A. thaliana* freeze protection

**Figure 10:**
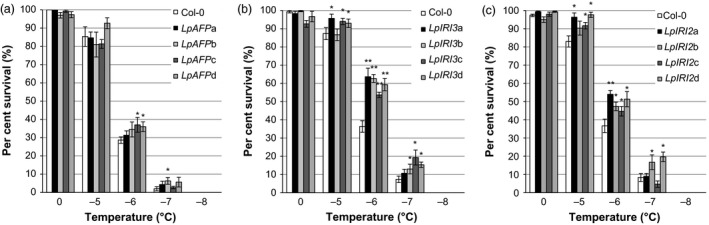
Freezing survival of transgenic *A. thaliana* expressing individual *Lp* IRIP isoforms. Shown are the survival rates of *LpAFP‐* (a) *LpIRI3‐* (b) and *LpIRI2‐* (c) expressing *A. thaliana* following recovery from temperatures between 0 and −8 °C. Transgenic plants were compared to nontransgenic *A. thaliana* plants (Col‐0). Error bars represent the standard errors of the mean and asterisks denote lines with significantly enhanced freezing survival compared with Col‐0 plants (*P < 0.05, **P < 0.005; two‐tailed *t*‐tests). Experiments were conducted using 100 seedlings for each independently generated transgenic line, in triplicate.

The addition of the various *Lp*IRIP‐bearing sequences dramatically enhanced the freeze survival of whole transgenic *A. thaliana* plants. Freeze survival was significantly increased compared with controls in two of the four *LpAFP‐*expressing lines following freezing at −6 °C (Figure 10a). In one of the *LpAFP* lines, there was also a significant increase in survival at −7 °C; however, the overall LT_50_ was not changed, remaining at −5.6 °C, not significantly different from the LT_50_ of −5.2 °C seen in control, nontransgenic plants. Significant increases in survival were seen in all four *LpIRI3*‐expressing lines at all tested temperatures between −5 °C and −7 °C (Figure 10b), and this was reflected in a mean LT_50_ of −6.1 °C. Similarly, *LpIRI2*‐transgenic *A. thaliana* showed enhanced freeze survival at all temperatures between −5 °C and −7 °C (Figure 10c). In these lines, the mean LT_50_ was −6.0 °C compared with −5.4 °C in control plants assayed at the same time.

**Figure 11:**
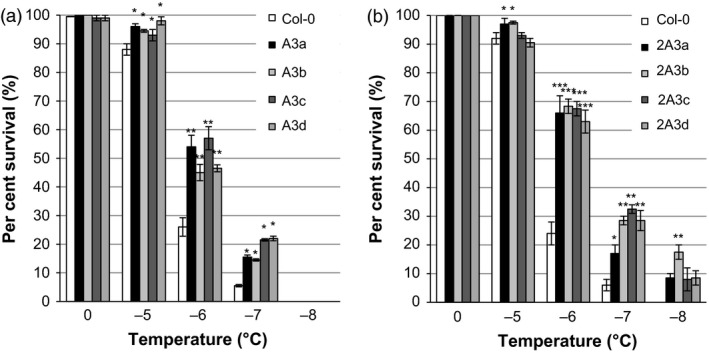
Survival rates of transgenic *A. thaliana* plants expressing multiple *Lp* IRIPs. Survival was calculated for *A. thaliana* plants expressing *LpAFP* and *LpIRI3* (A3) (a) and *LpIRI2*,* LpAFP* and *LpIRI3* (2A3) (b) following recovery of plants from temperatures between 0 and −8 °C. Error bars represent standard errors of the mean. Asterisks denote a significant increase in freezing survival (*P < 0.05, **P < 0.005, ***P < 0.0005; two‐tailed *t*‐tests) compared with Col‐0 lines. All experiments were performed using 100 seedlings per transgenic line, in triplicate.

Transgenic plants expressing multiple *Lp*IRIPs also showed enhanced freezing tolerance. Survival was significantly increased in both the A3 and 2A3 lines at temperatures between −5 °C and −8 °C (Figure 11a and b) with a concomitant significant decrease in LT_50_ to −6.0 °C and −6.4 °C, respectively, compared with the LT_50_ of −5.2 °C and −5.4 °C for the corresponding nontransgenic controls. Notably, none of the lines bearing a single *LpIRI* sequence or even the A3 lines showed any survival at −8 °C (Figures 10 and 11a), but each of the four 2A3 lines, with all three sequences, showed some survival (10%−17%) at this low temperature (Figure 11b).

## Discussion

Freezing tolerance is a complex trait involving biochemical, metabolic and physiological changes. In certain plants, IBPs almost certainly serve as part of a freeze survival strategy to regulate ice crystal growth and to lower the probability of plasma membrane rupture. These proteins have also been shown to lower the activity of bacterial ice nucleation, aiding in freeze survival (Tomalty and Walker, 2014). The *L. perenne* family of IBPs includes the ‘processed’ protein sequence, *Lp*AFP, which has been extensively characterized *in vitro* (Lauersen *et al*., 2011; Middleton *et al*., 2009); however, the *in planta* function and activity of the proteins transcribed and subsequently translated from the full‐length *Lp*IRIPs are less known. Here, our experiments demonstrate that in transgenic *A. thaliana,* the presence of *LpIRI2* and *LpIRI3* not only reduced electrolyte leakage but also significantly enhanced freeze survival (Figures 8, 9, 10, 11).

Importantly, the degree of freeze protection afforded by *Lp*IRIPs in *A. thaliana* was correlated with the ice‐binding activity localized to the apoplast (Figure 5). Most ice nucleation occurs outside of the cell (Kajava and Lindow, 1993; Xu *et al*., 1998). Intracellular ice nucleation typically only occurs as a result of rapid temperature drops (Siminovitch *et al*., 1978) and as temperatures were lowered slowly (0.5 °C/h), intracellular ice crystal growth would not be expected in these experiments. In this regard, it may be curious that in two *LpAFP*‐expressing lines, in which the ice activity appeared to have remained intracellular, there was a modest increase in freeze survival, suggesting that intracellular IBPs might additionally contribute to a freeze‐tolerant phenotype. Indeed, it is possible that due to the absence of extracellular IBPs and thus the increased probability of plasma membrane rupture, release of intracellular *Lp*AFP facilitated the protection of neighbouring cells in whole plants, explaining the increased survival of one of the *LpAFP* lines at −7 °C (Figure 10a). Nevertheless, the large increases in freezing stress survival rates observed in *LpIRI3‐*expressing *A. thaliana* lines underscore the dramatic freeze protection conferred by extracellular IBPs. In addition to restricting ice crystal growth, it has also been suggested that IBPs are capable of preventing cell lysis through physical association with the plasma membranes (Beirão *et al*., 2011; Hays *et al*., 1996; Rubinsky *et al*., 1991; Tomczak *et al*., 2001). Our results showing reduced electrolyte leakage in transgenic *LpIRI2* and *LpIRI3 A. thaliana* lines are consistent with the interpretation that membrane protection is occurring; however, there is no *in planta* evidence of a direct association between IBPs and plasma membranes.

Transgenic expression of multiple *Lp*IRIPs reduced ion leakage and increased freeze survival at lower temperatures than in many of the lines bearing a single *Lp*IRIP sequence (Figures 10 and 11). It is thus likely that multiple transgenes result in a greater accumulation of *Lp*IRIPs, even if this was not reflected in TH values given the hyperbolic relationship between TH activity and protein levels. However, the activity of *Lp*IRIPs can be distinguished on the basis of their adsorption to distinct ice crystal planes. Amino acid alignment suggests that the *Lp*IRI3 isoform has the most regular fold, and highest conservation amongst putative ice‐binding residues (Figure 1). In accordance with this observation, transgenic plants with the *LpIRI3* sequence either as a single copy, with *LpAFP*, or with *LpAFP* and *LpIRI2*, showed adsorption to both the primary prism and basal planes, characteristic of hyperactive AFPs (Pertaya *et al*., 2008). It is possible that there could be some synergistic activity with the different transgenes, resulting in reduced ice crystal growth when IBPs with slightly different ice plane affinities are combined. As well, IBPs could have differing affinities for any putative membrane‐binding sites, which in turn could enhance freeze survival.

These results are reminiscent of the enhancement of TH activity shown when a low‐activity type III AFP isoform was expressed in the Notched‐fin eelpout along with a high‐activity AFP isoform (Nishimiya *et al*., 2005). It was suggested that AFP isoforms might act cumulatively to enhance activity levels by high‐activity AFPs slowing the growth of ice crystals sufficiently so that less active AFPs would have time to adsorb, or alternatively, that less active AFPs could adsorb at ice crystal sites located between the binding sites of more active AFP isoforms (Nishimiya *et al*., 2005). Similarly, Burcham *et al*. (1984) hypothesized that antifreeze activity was enhanced in the presence of high‐ and low‐activity antifreeze glycoprotein (AFGP) isoforms, as a result of a cooperative coverage of the initial ice crystal. It is possible that *Lp*IRIPs may function similarly, restricting growth of the initial seed crystal more effectively when more than one isoform is present, providing optimal freeze protection to plants.

Putting aside possible isoform differences in ice or membrane substrate affinity, our results clearly demonstrate that the *Lp*IRI2 isoform with no identifiable signal sequence shows ice‐binding activity (TH, ice‐shaping and IRI) and CA accumulation in the apoplast, and can confer freeze protection to a host plant. Thus, the evolutionary loss of the N‐terminal domain does not render this protein nonfunctional. Similar transcript‐level estimates of *LpIRI2* and *LpIRI3* in CA *L. perenne* leaves, and the reduction in *A. thaliana* leaf electrolyte leakage*,* further support our contention that *LpIRI2* is not a pseudogene and could therefore play a role in *L. perenne* overwintering. As intracellularly localized IBPs are produced in certain plants including the desert evergreen shrub, *Ammopiptanthis mongolicus* (Fei *et al*., 1994), the flowering shrub, *Forsythia suspense* (Simpson *et al*., 2005)*,* and *S. dulcamara* (Duman, 1994) as well as the Antarctic microalga, *Chaetoceros neogracile* (Gwak *et al*., 2014), we initially suspected that *Lp*IRI2 functioned similarly. Of note, intracellularly localized IBPs in addition to restricting ice crystal growth have demonstrated comparably lower ice‐binding activity (Duman, 1994). Nonetheless, our experiments indicate that the *Lp*IRI2 isoform is secreted (Table 2 and Figures 5 and 7). Although levels of IRI and TH activities in the apoplast were admittedly lower than that observed with *LpIRI3*‐expressing lines, the fact that recombinant *Lp*IRI2 showed a reduced ability to restrict ice crystal growth compared with *Lp*IRI3 (Figure 2) suggests that such differences may be due to varying affinities for ice crystal planes or less efficient ice crystal adsorption. Attempts to localize this isoform, as well as other IBPs, using fluorescently tagged proteins *in planta* did not allow us to unambiguously distinguish between the intracellular plasma membrane and the apoplast (Supplementary 1). Nevertheless, IRI and ice‐shaping activity, as well as fluorescence, were present in apoplast extracts and guttation fluid of *LpIRI2‐*expressing *A. thaliana* lines (Figures 5 and 7, and Table 2). Additionally, the level of freeze tolerance observed in *LpIRI2* lines was superior to that seen for the intracellular *Lp*AFP lines, in accordance with extracellular localization. Therefore, taken together, these observations support the hypothesis that *Lp*IRI2 is a secreted protein.

Protein secretion using nonclassical pathways is a relatively unexplored area of research. Despite this, it has been estimated that 60% of proteins identified in the secretome of *A. thaliana* are leaderless secreted proteins (LSPs) (Regente *et al*., 2012). The mechanisms involved in the recognition of LSPs are not well known, and may not be conserved amongst or within secretion systems, suggesting that such mechanisms have evolved independently. Therefore, reliable prediction is not possible. Intriguingly, a nonclassical secretion system appears more common amongst protein families involved in environmental stress responses (Cheng *et al*., 2009; Gupta and Deswal, 2012; Kim *et al*., 2008). Thus, it is possible that non‐Golgi secretion could provide flexible spatial localization, allowing proteins to take on dual function roles inside and outside of the cell. Perhaps as important, given the large number of proteins up‐regulated during the freezing stress response, nonclassical secretion could provide an alternative, and potentially more efficient secretion for critical proteins under these circumstances, allowing other necessarily ER‐linked proteins to monopolize the ‘traditional’ pathway.

Secretion through the ER‐Golgi pathway is required for the post‐translational modification of proteins. In this regard, a number of putative glycosylation sites have been identified in *Lp*IRIP family members (Kuiper *et al*., 2001). Nevertheless, recombinantly produced *Lp*AFP retains IRI and TH activities, indicating that such modifications are not necessary for proper ice‐binding activity (Lauersen *et al*., 2011) and there has been no demonstration that *Lp*IRIPs are post‐translationally modified *in planta*. Further experiments regarding the localization of IBPs lacking classical secretion signals, as well as the possible nonclassical mechanisms of protein secretion, could prove useful in understanding the roles that IBPs play in plant freeze tolerance.

We believe that a lack of knowledge regarding the mechanisms underlying IBP‐induced freezing tolerance has hindered the development of freeze‐tolerant crops. In the past, a great deal of effort has been invested into the transfer of fish and insect AFPs to plants (e.g. Hightower *et al*., 1991; Holmberg *et al*., 2001; Huang *et al*., 2002; Kenward *et al*., 1999). These transgenes were considered attractive candidates given the ability of these proteins to depress the freezing point by several degrees. However, the expression of moderately TH‐active fish AFPs in plants has not yielded favourable results, likely due to the catastrophic needle‐like crystals that are formed once the freezing point is reached. These ice crystal burst patterns are due to adsorption of AFPs to the primary prism plane exclusively, resulting in growth from the c‐axis (Fletcher *et al*., 2001). Although insect AFPs direct the formation of hexagonal bipyramidal crystals, similar to those produced in the presence of plant IBPs, they have high TH activity. This could allow intracellular freezing at the same time or earlier than extracellular freezing, which would not be compatible with crop survival. We suggest that plant IBPs with their low TH activity, but relatively high IRI activity and ‘gentle’ burst morphologies (Middleton *et al*., 2012), should prove more efficacious for such applications. Certainly, given the inevitability that most overwintering plants will freeze, the use of IBPs that have evolved from a freezing tolerant survival strategy would logically appear to be more promising than AFPs that have evolved in species where freezing of the interstitial fluid is lethal. Here, we have shown that the expression of *Lp*IRIPs in *A. thaliana* produced a freeze‐tolerant phenotype that was enhanced in the presence of more than one isoform. These results strongly suggest that the expression of multiple *Lp*IRIP isoforms in a cold‐adapted but freeze‐susceptible crop may allow for even more substantial freezing tolerance capabilities than the striking ~2 °C seen here.

## Experimental procedures

### Bioinformatics analysis

The translated sequences corresponding to the open reading frames (ORFs) of *LpIRI1* (GenBank accession no. EU680848), *LpIRI2* (EU680849), *LpIRI3* (EU680850), *LpIRI4* (EU680851) and *LpAFP* (AJ277399) were aligned using ClustalW2 multiple sequence alignment tool (http://www.ebi.ac.uk/Tools/msa/clustalw2/). Predictions regarding the IRI‐domain and putative ice‐binding residues for *Lp*IRIP sequences were made based on alignment with *Lp*AFP, for which the IBF has been well characterized (Middleton *et al*., 2009). SignalP 4.1 (http://www.cbs.dtu.dk/services/SignalP/) was used to predict sequences encoding putative signal peptides. The Phyre 2.0 algorithm (http://www.sbg.bio.ic.ac.uk/phyre2/html/page.cgi?id=index) was used to predict secondary protein structure.

### Cloning and protein purification of recombinant LpIRIPs

Sequences corresponding to the ORFs of *LpIRI2* and *LpIRI3* were synthesized by GeneArt^™^ (Invitrogen, Carlsbad, CA), and the stop codons were removed by PCR with the primers LpIRI2NdeIFW/LpIRI2nostopXhoIRV (Table 3a) for *LpIRI2* and LpIRI3NdeIFW/LpIRI3nostopXhoIRV (Table 3a) for *LpIRI3*, in order to incorporate a 6‐residue histidine tag to facilitate protein purification. Amplification was performed using Platinum Pfu Taq DNA polymerase (Invitrogen, Carlsbad, CA) using the following program: 2 min at 94 °C followed by 35 cycles of 30 sec at 94 °C, 30 s at 54 °C and 1 min 50 s at 72 °C, with a final extension at 72 °C for 7 min. The amplified products were then ligated into pET24a(+) vectors (Novagen, Etobicoke, ON, CA) and transformed into ArcticExpress^™^ *Escherichia coli* cells (New England Biolabs Inc., Whitby, ON, CA) using chemical transformation, with each construct subsequently confirmed by sequencing (Plateforme de séquençage et de génotypage des genomes; Québec City, QC, CA) after each cloning step.

**Table 3:** Primer sequences used for the cloning of *Lp*IRIP constructs prior to transformation of *A. thaliana* plants (a) and reverse transcription PCR analysis of *LpIRI2* and *LpIRI3* transcripts (b), with underlined sequences correspond to internal restriction sites used for cloning purposes

	Primer	Primer sequence	Tm (°C)
(a) Construct			
pET24a(+):*LpIRI2*:His	LpIRI2NdeIFW	5′‐TTAACATATGCCATTACATGTGAAGCG‐3′	58
	LpIRI2nostopXhoIRV	5′‐TTAACTCGAGACCTCCTGTCACGACTTTG‐3′	58
pET24a(+):*LpIRI3*:His	LpIRI3NdeIFW	5′‐TTAACATATGGCGAAATGCTTGATGCT‐3′	58
	LpIRI3nostopXhoIRV	5′‐TTAACTCGAGAGCGTCTGTCACGACTTTG‐3′	58
pCAMBIA1305.1:*LpAFP*	LpAFPBglIIFW	5′‐TTAAAGATCTTATGGATGAACAGCCGAAA‐3′	64
	LpAFPPmlIRV	5′‐TTAACACGTGTTAAGCGTCTGTCACGACT‐3′	76
pCAMBIA1305.1:*LpIRI2*	LpIRI2BglIIFW	5′‐TTAAAGATCTTATGCCATTACATGTGAAGCG‐3′	58
	LpIRI2PmlIRV	5′‐TTAACACGTGTTAACCTCCTGTCACGACTT‐3′	58
pCAMBIA1305.1:*LpIRI3*	LpIRI3BgIIIFW	5′‐TTAAAGATCTTATGGCGAAATGCTTGATGCT‐3′	58
	LpIRI3PmlIRV	5′‐TTAACACGTGTTAAGCGTCTGTCACGACTT‐3′	58
pCAMBIA1305.1:*LpAFP*‐*LpIRI3*	35SBamHIFW	5′‐TTTAAGGATCCCATGGAGTCAAAGATTCAAATAG‐3′	62
	NOSterHindIIIRV	5′‐TTAAAAGCTTGTTTACCCGCCAATATATCCT‐3′	60
pCAMBIA1305.1:*LpIRI2*‐*LpAFP*‐*LpIRI3*	35SEcoRIFW	5′‐TTTAAGAATTCCATGGAGTCAAAGATTCAAATAG‐3′	62
	NOSterSacIRV	5′‐TTAAGAGCTCGTTTACCCGCCAATATATCCT‐3′	60
pCAMBIA1305.1:*mORANGE*‐*LpAFP*	OFPBglIIFW	5′‐AATTAGATCTTATGGTGAGCAAGGGCGAGG‐3′	65
	LpAFPPmlIRV	5′‐AATTCACGTGTTAAAGCTTTGCAGCGTCTGTCACG‐3′	65
pCAMBIA1305.1:*LpIRI2*‐*mORANGE*	LpIRI2NcoIFW	5′‐TTAACCATGGTTATGCCATTACATGTGAAGCG‐3′	58
	LpIRI2nostopBglIIRV	5′‐TTAAAGATCTGTACCTCCTGTCACGACTTTG‐3′	58
pCAMBIA1305.1:*LpIRI3*‐*mORANGE*	LpIRI3NcoIFW	5′‐TTAACCATGGTTATGGCGAAATGCTTGATGCT‐3′	58
	LpIRI3nostopBglIIRV	5′‐TTAAAGATCTGTAGCGTCTGTCACGACTTTG‐3′	58
(b) Transcript			
*LpIRI2*	LpIRI2BglIIFW	5′‐TTAAAGATCTTATGCCATTACATGTGAAGCG‐3′	58
	LpIRI2PmlIRV	5′‐TTAACACGTGTTAACCTCCTGTCACGACTT‐3′	58
*LpIRI3*	LpIRI3BgIIIFW	5′‐TTAAAGATCTTATGGCGAAATGCTTGATGCT‐3′	58
	LpIRI3PmlIRV	5′‐TTAACACGTGTTAAGCGTCTGTCACGACTT‐3′	58

Bacterial cultures were grown to an optical density (OD) of 0.8 (λ = 595) and induced for 48 h at 16 °C, using 0.5 mm of isopropyl β‐D‐1‐thiogalactopyranoside (IPTG). Cells were lysed using a French Press (ThermoFisher Scientific, Nepean, ON, CA) and recombinant proteins purified from soluble lysates using a nickel‐NTA agarose column (Qiagen, Toronto, ON, CA) as previously described (Lauersen *et al*., 2011). Purified proteins were dialysed against 50 mm Tris–HCl and 100 mm NaCl, pH 8.0, for 24 h and used immediately for ice‐binding and protein assays or frozen at −20 °C until analysed.

### Cloning of *Lp*IRIP constructs for expression in *A. thaliana*

For expression in *A. thaliana,* the ORFs of *LpAFP*,* LpIRI2* and *LpIRI3* were PCR‐amplified using LpAFPBglIIFW/LpAFPPmlIRV, LpIRI2BglIIFW/LpIRI2PmlIRV and LpIRI3BglIIFW/LpIRI3PmlIRV primers, respectively (Table 3a) with the following protocol: 95 °C for 2 min followed by 35 cycles of 95 °C for 45 s, 50 °C for 45 s and 72 °C for 1 min, with a final extension of 7 min at 72 °C. All fragments were ligated into pCAMBIA1305.1 vectors (Cambia, Canberra, ACT, AU) under the control of a cauliflower mosaic virus (CaMV) 35S promoter and a nopaline synthase (NOS) terminator. The construct for the simultaneous expression of *LpIRI3* and *LpAFP* (A3) was generated by PCR amplification of 35S:*LpAFP*:NOS with 35SBamHIFW/NOSterHindIIIRV primers (Table 3a), using the protocol described above, and inserting the amplified fragment into the pCAMBIA1305.1:*LpIRI3* construct. Subsequently, 35S:*LpIRI2*:NOS was amplified using 35SEcoRIFW/NOSterSacIRV (Table 3a) and inserted into the A3 construct for expression of all three isoforms (2A3), again using the same program.

The gene sequence corresponding to *mOrange*‐*LpAFP* was ligated into pCAMBIA1305.1 following PCR amplification using OFPBglIIFW/LpAFPPmlIRV primers (Table 3a) under the following thermocycler program: 94 °C for 2 min, followed by 35 cycles of 94 °C for 30 s, 57 °C for 30 s and 72 °C for 1 min 30 s with a final extension of 72 °C for 10 min. *LpIRI2* and *LpIRI3* were amplified using LpIRI2NcoIFW/LpIRI2nostopBglIIRV and LpIRI3NcoIFW/LpIRI3nostopBglIIRV primers (Table 3a), respectively, using the PCR conditions described for recombinant *Lp*IRIP constructs, and inserted upstream of the mOrange tag to avoid disruption of the N‐terminal signal sequence. In contrast, by placing the mOrange tag upstream of *LpAFP*, we could ensure that this protein remained intracellular, serving as control for cytoplasmic localization. Gene sequences were again confirmed by sequencing.

### Plant materials and growth conditions

*Lolium perenne* seeds (Pacific Seed diploid variety; Premier Specific Seeds, Surrey, BC, CA) used for transcript analysis were grown in potting soil and maintained in a growth chamber (Queen's University, Kingston, ON, CA) on a 20‐h/4‐h light/dark cycle at 22 °C/18 °C with humidity and light regulated at 70% and 175 μmol/m^2^/s, respectively. Prior to reverse transcription (RT) PCR analysis, plants were grown for 3 weeks prior to a 6‐day acclimation period at 21 °C (no light; control) or a cold acclimation period at 4 °C (no light).

All transgenic expression experiments were conducted using wild‐type *A. thaliana* (ecotype: Col‐0). For crude cell extractions, apoplast extractions, collection of guttation fluid and electrolyte leakage assays, *A. thaliana* were sown in potting soil and grown in a growth chamber for 3 weeks under standard growth conditions with a 16‐h/8‐h light/dark cycle at 22 °C/20 °C, 70% relative humidity, and light ~150 μmol/m^2^/s. Seeds used for survival assays were surface‐sterilized using 70% (v/v) ethanol with 0.05% (v/v) Triton X‐100 for 5 min, followed by a 95% (v/v) ethanol wash for 5 min, and plated on 0.5× Murashige and Skoog (MS) agar prior to transfer to standard growth conditions.

Prior to experimentation, plants were cold‐acclimated (CA) at 4 °C, on a short‐day cycle (6‐h/18‐h light/dark), with ~175 μmol/m^2^/s light for 48 h. These conditions were imposed for two reasons: first, we sought to prevent *Lp*IRIP misfolding, which occurs above 16 °C (Lauersen *et al*., 2011), and secondly, we wished to limit the time at 4 °C, because *A. thaliana* reaches optimal freezing tolerance after 1 week of low‐temperature exposure (Uemura *et al*., 1995). Thus, we attempted to balance the accumulation period of well‐folded *Lp*IRIPs and at the same time reduce the possible confounding effect of up‐regulating other cold‐induced freezing tolerance host mechanisms.

### Generation of *A. thaliana* transgenic lines

Tagged and untagged *Lp*IRIP constructs designed for expression in *A. thaliana* were transformed into GV3101 *Agrobacterium tumefaciens* cells by electroporation. *Agrobacterium*‐mediated transformation of *A. thaliana* was carried out using the floral dip method (as described in Middleton *et al*., 2014). Successfully transformed plants were selected on 0.5× MS medium plates containing hygromycin (50 μg/mL). Four independent, homozygous lines were generated for each construct.

### Endogenous *Lp*IRIP transcript analysis

RNA was collected from the leaf tissue of CA *L. perenne* grown under the conditions described above. Extractions were performed using the RNeasy Plant Mini Kit (Qiagen, Toronto, ON, CA) followed by cDNA synthesis using Superscript^®^ III First‐Strand Synthesis System (Invitrogen, Carlsbad, CA) according to the manufacturers’ specifications. RT‐PCR was carried out using the following cycle conditions: 95 °C for 5 min, 94 °C for 30 s, 53 °C for 30 s and 72 °C for 1 min, for 45 cycles. *LpIRI2* was amplified using LpIRI2BglIIFW/LpIRI2PmlIRV primers (Table 3b) and *LpIRI3* was amplified using LpIRI3BgIIIFW/LpIRI3PmlIRV primers (Table 3b). A ‘housekeeping gene’, s‐adenosylmethionine decarboxylase transcript (*SamDC*), was amplified with primers as previously described (Hong *et al*., 2008) using the following cycle conditions: 95 °C for 2 min, followed by 35 cycles of 94 °C for 30 s, 55 °C for 30 s and 72 °C for 30 s.

### Ice‐binding and protein assays

Ice‐binding assays were performed using protocols that have been optimized for plant IBPs as described previously. IRI activity was assessed using splat assays and ice‐shaping activity and TH activity assayed using a nanolitre osmometer (Middleton *et al*., 2014). Prior to analysis, crude cell lysates (Lauersen *et al*., 2011), apoplast extracts (Villers and Kwak, 2013) and guttation fluid (Madsen *et al*., 2016) were prepared from 4‐week‐old CA *A. thaliana* leaf tissue as described previously. Plant extracts were suspended in a native protein extraction buffer (10 mm Tris–HCl, 25 mm NaCl, pH 7.5). Recombinant proteins used for ice‐binding assays were prepared as described above. Protein concentration was determined using the Pierce^™^ BCA Protein Assay Kit (ThermoFisher Scientific, Nepean, ON, CA) following the manufacturer's instructions, with all experiments carried out in triplicate.

### Electrolyte leakage assay

Electrolyte leakage assays using 4‐week‐old CA *A. thaliana* plants were carried out using a modified protocol from Thalhammer *et al*. (2014). Two mature rosette leaves were cut and placed in glass tubes containing deionized water (100 μL). Treatments were conducted by placing tubes in a circulating water bath at 0 °C and lowering the temperature to −1 °C, over 30 min. Samples were then nucleated using a single ice chip, and the temperature was decreased (1 °C every 15 min) to −6 °C. Following treatment, experimental samples were removed from the circulating water bath and allowed to recover overnight at 4 °C in the dark. Control samples were left covered in the dark at 4 °C, while treated experimental samples were prepared and allowed to recover at 4 °C overnight. All cut leaf samples were then transferred to conical tubes (50 mL containing 20 mL of deionized water) and shaken for 18 h at 24 °C, 150 rpm. Initial (C_i_) and final conductivity (C_f_) measurements were taken before and after autoclaving samples using a direct reading conductivity meter (Bach‐Simpson Ltd., London, ON, CA) and presented as a percentage (100 C_i_C_f_^−1^) with 12 individual plants for each independent line, in triplicate. Significance was evaluated using two‐tailed *t*‐tests (*P* < 0.05).

### *A. thaliana* freeze survival assay

Whole plant freeze survival assays were modified from Xin and Browse (1998). *A. thaliana* seedlings (~2000 per experiment) were grown for 4 weeks on MS agar plates rather than soil to reduce the presence of any ice‐nucleating bacteria. Plates were then transferred to −1 °C (no light) for 2 h prior to nucleation using an ice chip in the centre of the plate and kept at this temperature for 12 h to ensure that the agar was frozen. The temperature was then lowered by 1 °C every 2 h, until the temperature reached −8 °C. One plate for each line was removed at temperatures indicated in the Results (between −5 and −8 °C) and allowed to recover at 4 °C (no light) for 24 h, prior to transfer to standard growth conditions for 14 days, when per cent survival and LT_50_ were calculated. Assays were performed using 100 seedlings per independent line, in triplicate, with significant differences evaluated using a two‐tailed *t*‐test (*P* < 0.05).

### Fluorescence readings

The level of mORANGE‐tagged protein present in the apoplast extracts of transgenic *A. thaliana* plants was determined using the SpectraMax Gemini XS microplate reader (Molecular Devices, Sunnyvale, CA). Emission and excitation wavelengths of 562 nm and 548 nm, respectively, were used based on the specifications of the mORANGE protein product. Readings were taken at a total protein concentration of 0.1 mg/mL.

### Confocal microscopy

Two‐week‐old seedlings grown on 0.5× MS agar plates were cold‐acclimated for 48 h at 4 °C (6 h light) prior to visualization. A laser scanning microscope (LSM710; Ziess, Oberkochen, BW, DE) was used to visualize mOrange‐tagged proteins (~543 nm). Images were obtained using ZEN 2009 software.

## Supporting information


**Figure S1** Expression of fluorescently tagged *Lp*IRIP constructs in the roots of transgenic *A. thaliana* plants. Roots were visualized using a confocal microscope. Experiment was performed in duplicate.
