# Supplementary material for: Ice‐binding proteins confer freezing tolerance in transgenic Arabidopsis thaliana
Source: Plant Biotechnol J. 2016 Jul 14;15(1):68–81. doi: 10.1111/pbi.12592 (PMC5253476; doi:10.1111/pbi.12592)
Supplement: Supplementary file 1 — Figure S1 Expression of fluorescently tagged LpIRIP constructs in the roots of transgenic A. thaliana plants. Roots were visualized using a confocal microscope. Experiment was performed in duplicate. [file PBI-15-68-s001.docx]

**Supporting Information**


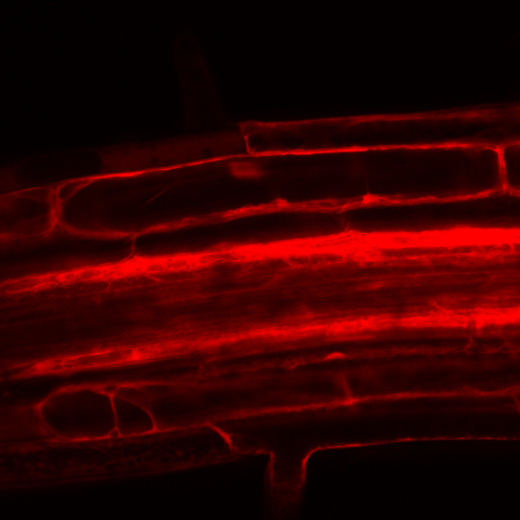


mOrange


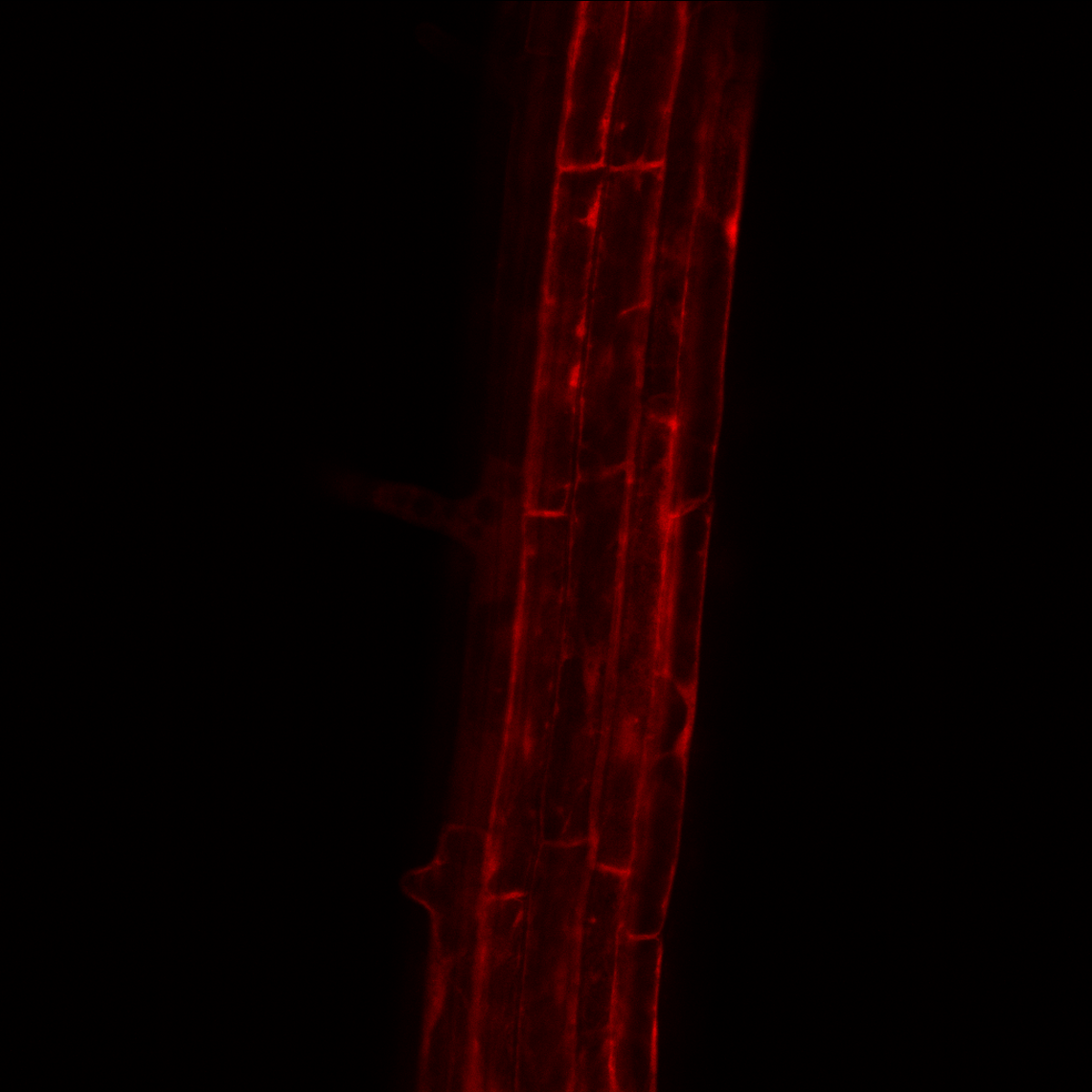


*Lp*IRI3-mOrange


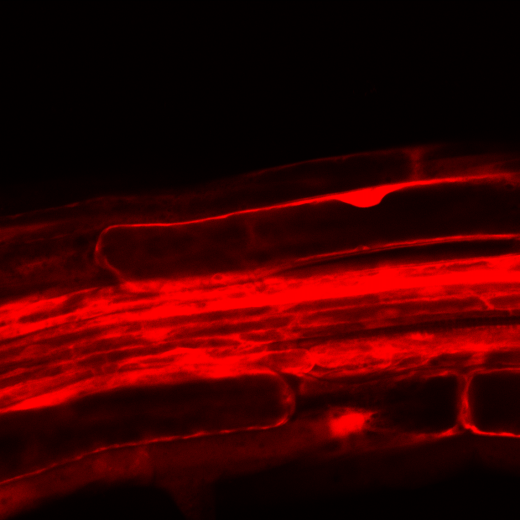


mOrange-*Lp*AFP


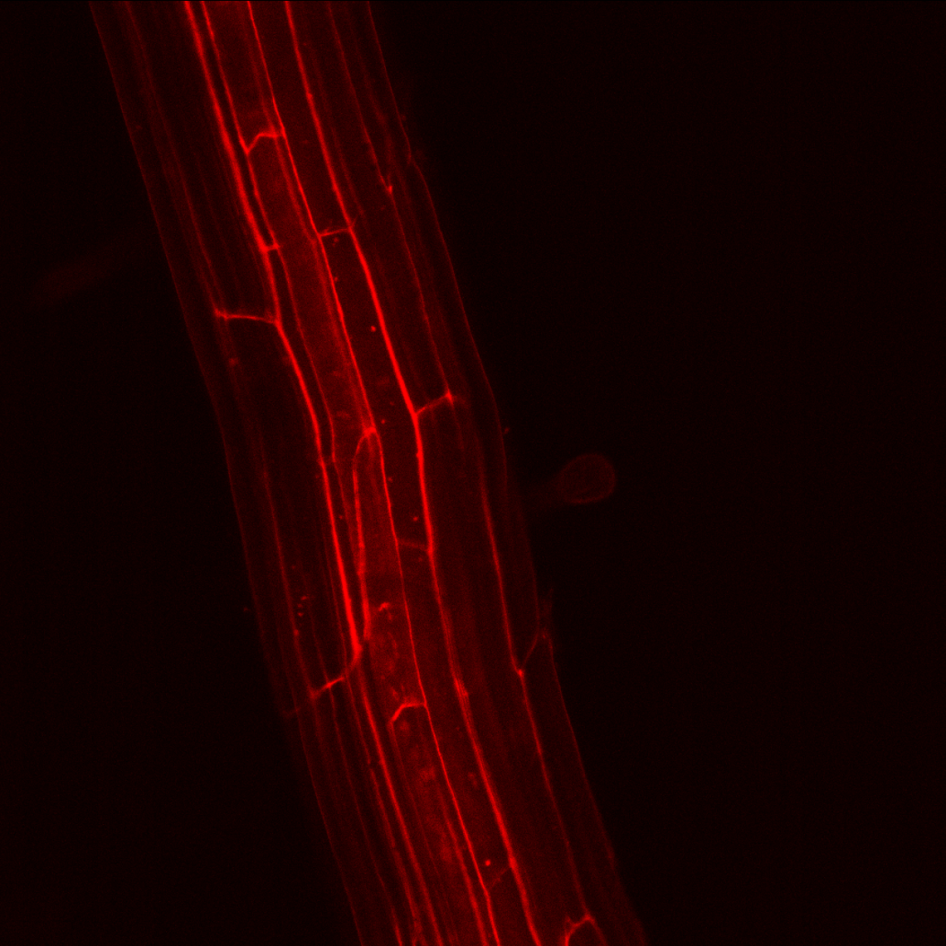


*Lp*IRI3-mOrange

**Supplementary Figure 1.** Expression of fluorescently-tagged *Lp*IRIP constructs in the roots of transgenic *A. thaliana* plants. Roots were visualized using a confocal microscope. Experiment was done in duplicate.
